# Prognostic Value of YTHDF2 in Clear Cell Renal Cell Carcinoma

**DOI:** 10.3389/fonc.2020.01566

**Published:** 2020-09-23

**Authors:** Zhongyi Mu, Dan Dong, Mingli Sun, Liwen Li, Ning Wei, Bin Hu

**Affiliations:** ^1^Department of Urology, Cancer Hospital of China Medical University, Liaoning Cancer Hospital & Institute, Shenyang, China; ^2^Department of Pathophysiology, College of Basic Medical Science, China Medical University, Shenyang, China; ^3^School of Kinesiology, Shenyang Sport University, Shenyang, China; ^4^Department of Biostatistics, Fairbanks School of Public Health, Indiana University, Indianapolis, IN, United States; ^5^Division of Hematology-Oncology, Department of Medicine, University of Pittsburgh School of Medicine, Pittsburgh, PA, United States; ^6^Cancer Therapeutics Program, UPMC Hillman Cancer Center, University of Pittsburgh, Pittsburgh, PA, United States

**Keywords:** m6A-related genes, The Cancer Genome Atlas, prognosis, YTHDF2, clear cell renal cell cancer

## Abstract

m6A, the main form of mRNA modification, participates in regulating multiple normal and pathological biological events, especially in tumorigenesis. However, there is little known about the association of m6A-related genes with prognosis of clear cell renal cell cancer (ccRCC). Therefore, the prognostic value of m6A-related genes was investigated using Kaplan–Meier curves of overall survival (OS) with the log-rank test and Cox regression analysis. The differential expression of YTHDF2 mRNA in ccRCC and tumor-adjacent normal tissues and associated with clinicopathological characteristics was also analyzed. The alteration of cancer signaling pathways was screened by Gene Set Enrichment Analysis (GSEA). Univariate analysis showed that 15 m6A-related genes (including YTHDF2) were closely related to prognosis. Multivariate analysis further confirmed that YTHDF2 could serve as an independent prognostic factor for the OS of ccRCC patients (*P* < 0.001). Low-level expression of YTHDF2 had poor prognosis in ccRCC patients with lower tumor–node–metastasis (TNM) stage, age > 61, non-distant metastasis, non-lymph node metastasis, female gender, and higher histological grade (*P* < 0.05). Moreover, YTHDF2 expression in ccRCC tissues (*N* = 529) is significantly lower than that of tumor-adjacent normal tissues (*N* = 72, *P* = 0.0086). Furthermore, GSEA demonstrated that AKT/mTOR/GSK3 pathway, EIF4 pathway, CHREBP2 pathway, MET pathway, NFAT pathway, FAS pathway, EDG1 pathway, and CTCF pathway are altered in tumors with high YTHDF2 expression. Taken together, our results demonstrated that YTHDF2 (an m6A-related gene) could serve as a potential prognostic biomarker of ccRCC, and targeting epigenetic modification may be a novel therapeutic strategy for the treatment of ccRCC.

## Introduction

In 2018, 65,340 new cases of renal cell carcinoma (RCC) were diagnosed and 14,970 resulted deaths in the United States (1). Moreover, ~3/4 of RCC belongs to clear cell renal cell cancer (ccRCC) (2). There are three main treatment measures for ccRCC, including radiotherapy, chemotherapy, and surgical resection (3–5). Due to the developments in medical imaging, the accurate for diagnostic rate of ccRCC is increased. However, ~30% of patients have distant metastasis once diagnosed (6), and these patients cannot be suitable for resection. Currently, the primary therapeutic measure for metastatic RCC (mRCC) is antiangiogenic therapy-based targeting tyrosine kinase. Although this treatment is of benefit for mRCC patients, and the reason for limited efficacy is development of drug resistance (7, 8), this biochemical alteration leads to poorer prognosis (9). Therefore, understanding the precise mechanisms of mRCC and looking for the key clinical biomarker and therapeutic target for RCC metastasis will contribute to successful treatment ccRCC.

*N*^6^-Methyladenosine (m6A), a key modification event of RNA, manipulated a series of genes called “writers” (METTL3, METTL14, and WTAP), “readers” (YTHDF1, YTHDF2, YTHDF3, YTHDC1, and YTHDC2), and “erasers” (FTO and ALKBH5) (10). In general, m6A is near the long internal exons and stop codons, located in 3′-UTRs (11), resulting in changes of RNA stability, splicing, intracellular distribution, and translation (12–14). Recently, several studies reported that modification of m6A exerted a key role in multiple tumorigenesis (15–17). Previous studies demonstrated that genetic alterations of m6A-mediated genes occurred in ccRCC. The alteration of m6A-mediated genes is closely associated with poor clinical characteristics, including overall survival (OS) (18). In addition, METTL3, as a tumor suppressor, plays a crucial role in process of proliferation, migration/invasion, and cell cycle regulation of ccRCC cells (19). To date, there is little known about the correlation of m6A-related genes profile and clinicopathological character of ccRCC. Thus, the prognostic value of m6A-related genes was investigated using Kaplan–Meier curve with Cox regression analysis and log-rank test. The differential YTHDF2 expression in ccRCC and tumor-adjacent normal tissues and associated with clinicopathological characteristics was analyzed by using mRNA expression data from The Cancer Genome Atlas (TCGA) ccRCC cohort. The alteration of multiple cancer signaling pathways were identified by Gene Set Enrichment Analysis (GSEA).

## Materials and Methods

### RNA-Seq Gene Expression Analysis in Clear Cell Renal Cell Cancer Patients

Based on the TCGA (https://tcga-data.nci.nih.gov/tcga/) data portal, m6A-related gene expression data (HTSeq-FPKM data) and clinicopathological features of 529 ccRCC patients and 72 tumor-adjacent normal renal samples were obtained. The clinicopathological characteristics of ccRCC patients are listed in Table 1, as follows: age, sex, tumor grade, tumor–node–metastasis (TNM) stage, cancer status, laterality, race, hemoglobin, platelet, serum calcium, and white blood cell (WBC) count. The patients without complete clinicopathological characteristics were excluded. The repeat gene expression data for the same patient were averaged. Finally, the correlation of RNA-Seq gene expression of 529 ccRCC patients and clinic information were investigated.

**Table 1:** Clinical characteristics of The Cancer Genome Atlas (TCGA) clear cell renal cell cancer (ccRCC) patients.

**Clinical features**	**Category**	**Overall survival**				
		**Patients**	**MST (days)**	**No. of events**	**HR(95% CI)**	***P-*value**
Age	>61/ ≤ 61	248/281	1,111/1,274	102 (41.1%)/70 (24.9%)	1.766 (1.302–2.394)	*<0.001*
Sex	Male/Female	343/186	1,170/1,204	110 (32.1)/62 (33.3%)	0.955 (0.700–1.304)	0.774
T stage	T3+T4/T1+T2	190/339	960/1,317	103 (54.2%)/69 (20.4%)	3.152 (2.322–4.277)	*<0.001*
N stage	N_1_/N_0_	16/238	456/1,303	10 (62.5%)/84 (35.3%)	3.079 (1.590–5.963)	*0.001*
M Stage	M_1_/M_0_	80/439	678/1,308	65 (81.3%)/106 (24.1%)	4.413 (3.237–6.016)	*<0.001*
Histological grade	G_3_+G_4_/G_1_+G_2_	281/240	1,126/1,285	128 (45.6%)/43 (17.9%)	2.715 (1.921–3.837)	*<0.001*
TNM stage	III-IV/I-II	205/321	952/1,371	114 (55.6%)/57 (17.8%)	3.770 (2.742–5.183)	*<0.001*
Cancer status	With tumor/Without tumor	138/357	948.5/1,343	103 (74.6%)/56 (15.7%)	5.390 (3.891–7.466)	*<0.001*
Laterality	Right/Left	279//249	1,238/1,133	77 (27.6%)/95 (38.2%)	0.689 (0.510–0.931)	*<0.015*
Race	Asian and Black/White	64/458	58.2/1,291	12 (18.8)/160 (34.9%)	0.824 (0.457–1.485)	0.520
Hemoglobin	Low/Normal	261/183	1,092/1,398	116 (44.4%)/41 (22.4%)	2.310 (1.615–3.305)	*<0.001*
Platelet	Low/Normal	44/358	1,194/1,238	19 (43.2%)/106 (29.6%)	1.667 (1.022–2.721)	0.041
Serum calcium	Low/Normal	203/150	1,133/1,184	62 (32.0%)/61 (40.7%)	0.778 (0.549–1.104)	0.160
WBC	Elevated/Normal	163/266	1,133/1,228	45 (27.6%)/102 (38.3%)	0.757 (0.532–1.076)	0.120

*TCGA, The Cancer Genome Atlas; ccRCC, Clear cell renal cell carcinoma; TNM stage, tumor, node, metastasis stage; MST, Median Survival Time. Italic values represent statistical significance*.

### Gene Set Enrichment Analysis

According to the cutoff value of YTHDF2 gene, all the ccRCC samples were defined as high- and low-level expression groups. GSEA was obtained from the GSEA program from sangerbox software (http://sangerbox.com/) using the BioCarta gene profile. Meanwhile, three factors [normalized enrichment score (NES), nominal *P*-value (NOM *P*-val), and false discovery rate (FDR)] were evaluated for statistical significance and enrichment magnitude (20).

### Statistical Analysis

X-tile software (Version 3.6.1) (21) was used to determine the optimal cutoff values for expression profile of m6A-related genes. Mann–Whitney test was used to analyze the expression difference between ccRCC samples and tumor-adjacent normal tissues. Chi-square test was used to evaluate the association between YTHDF2 and each clinicopathological characteristics of ccRCC patients, ignoring the effect of the other characteristics (shown as *P*-value). Multiple logistic regression model and Wald test were used to determine the association between YTHDF2 and each clinicopathological characteristic of ccRCC patients, adjusting for the effect of the other characteristics (shown as adjusted *P*-value). Log-rank test and Kaplan–Meier curve were used to compare the survival times. According to Cox hazards regression (HR) model, univariate and multivariate survival analyses were used to analyze the independent parameters associated with the OS. Prism software (Version 6.0), SPSS (Version 16.0), and SAS (Version 9.4) were used to perform data statistics. *P* < 0.05 was considered to be statistically significant.

## Results

### Clinical Profile and Prognosis of All Clear Cell Renal Cell Cancer Patients

The correlation of clinicopathological characteristic with the OS of 529 ccRCC patients was analyzed by univariate Cox proportion model. As shown in Table 1, age (>61/ ≤ 61), T stage (T3+T4/T1+T2), N stage (N_1_/N_0_), M stage (M_1_/M_0_, histological grade (G_3_+G_4_/G_1_+G_2_), cancer status (with/without tumor), TNM stage (III–IV/I–II), laterality (right/left), hemoglobin level (low/normal), and platelet level (low/normal) were closely related to the OS (*P* < 0.05), while sex (male/female), race (Asian and Black/White), serum calcium level (low/normal), and WBC (elevated/normal) were not significantly associated with the OS (*P* > 0.05) in patients with ccRCC.

### Identification of m6A-Related Gene YTHDF2 as a Prognostic Factor in Clear Cell Renal Cell Cancer

Univariate Cox proportion model and calculation of hazard ratio were performed to screen prognostic factors from a total of 19 m6A-related genes. As shown in Table 2, 15 m6A-related genes (including ALKBH, FTO, METTL3, METTL14, YTHDF2, YTHDC1, YTHDC2, ZC3H13, METTL16, KIAA1429, CBLL1, IGF2BP1, IGF2BP2, IGF2BP3, and RBM15) were significantly correlated to prognosis of ccRCC (*P* < 0.05). Furthermore, multivariate Cox regression model revealed that YTHDF2 (HR = 0.471, 95% CI: 0.241–0.920; *P* = 0.028) as well as age (HR = 2.118, 95% CI: 1.142–3.931; *P* = 0.017) and cancer status (HR = 3.329, 95% CI: 1.608–6.526; *P* = 0.001) served as independent prognostic factors (*P* < 0.05).

**Table 2:** Univariate and multivariate analysis of overall survival using the Cox proportional hazards regression model.

**Variables**	**Category**	**Univariable analysis**		**Multivariable analysis**	
		**HR (95% CI)**	***P*-value**	**HR (95% CI)**	***P-*value**
Age	>61/ ≤ 61	1.766 (1.302–2.394)	<0.001	2.118 (1.142–3.931)	**0.017**
Sex	Male/Female	0.955 (0.700–1.304)	0.774	–	–
T Stage	T3+T4/T1+T2	3.152 (2.322–4.277)	<0.001	1.101 (0.373–3.255)	0.861
N Stage	N_1_/N_0_	3.079 (1.590–5.963)	0.001	0.632 (0.155–2.569)	0.632
M Stage	M_1_/M_0_	4.413 (3.237–6.016)	<0.001	2.207 (0.912–5.341)	0.079
Histological grade	G_3_+G_4_/G_1_+G_2_	2.715 (1.921–3.837)	<0.001	1.571 (0.752–3.281)	0.230
TNM stage	III–IV/I–II	3.770 (2.742–5.183)	<0.001	1.083 (0.309–3.799)	0.901
Cancer status	With tumor/Without tumor	5.390 (3.891–7.466)	*<0.001*	3.329 (1.608–6.526)	**0.001**
Laterality	Right/Left	0.689 (0.510–0.931)	*<0.015*	1.250 (0.673–2.322)	0.479
Race	Asian and Black/White	0.824 (0.457–1.485)	0.520	–	–
Hemoglobin	Low/Normal	2.310 (1.615–3.305)	*<0.001*	1.288 (0.666–2.489)	0.452
Platelet	Low/Normal	1.667 (1.022–2.721)	0.041	0.991 (0.420–2.335)	0.983
Serum calcium	Low/Normal	0.778 (0.549–1.104)	0.160	–	–
WBC	Elevated/Normal	0.757 (0.532–1.076)	0.120	–	–
ALKBH5	Low/high (cutoff = 33.8)	0.668 (0.465–0.957)	0.028	1.119 (0.542–2.307)	0.762
FTO	Low/high (cutoff = 8.4)	0.517 (0.383–0.698)	<0.001	0.914 (0.418–1.999)	0.821
METTL3	Low/high (cutoff = 7.3)	2.216 (1.465–3.352)	<0.001	1.185 (0.450–3.119)	0.731
METTL14	Low/high (cutoff = 2.9)	0.452 (0.333–0.612)	<0.001	1.226 (0.471–3.192)	0.677
WTAP	Low/high (cutoff = 17.3)	1.356 (0.952–1.932)	0.092	–	–
YTHDF1	Low/high (cutoff = 15.6)	1.267 (0.939–1.710)	0.121	–	–
YTHDF2	Low/high (cutoff = 15.8)	0.605 (0.448–0.817)	0.001	0.471 (0.241–0.920)	**0.028**
YTHDF3	Low/high (cutoff = 14.0)	0.833 (0.618–1.124)	0.232	–	
YTHDC1	Low/high (cutoff = 11.5)	0.582 (0.430–0.787)	<0.001	0.938 (0.422–2.082)	0.874
YTHDC2	Low/high (cutoff = 4.5)	0.653 (0.476–0.896)	0.008	1.046 (0.542–2.021)	0.893
ZC3H13	Low/high (cutoff = 6.1)	0.479 (0.355–0.647)	<0.001	1.031 (0.447–2.377)	0.943
METTL16	Low/high (cutoff = 4.6)	0.588 (0.411–0.840)	0.004	0.499 (0.224–1.112)	0.089
KIAA1429	Low/high (cutoff = 5.3)	0.595 (0.441–0.803)	0.001	0.559 (0.235–1.328)	0.187
CBLL1	Low/high (cutoff = 4.6)	0.527 (0.388–0.715)	<0.001	1.261 (0.441–3.609)	0.666
IGF2BP1	Low/high (cutoff = 0.0)	1.841 (1.166–2.908)	0.009	0.853 (0.325–2.236)	0.747
IGF2BP2	Low/high (cutoff = 1.6)	2.309 (1.666–3.199)	<0.001	1.431 (0.554–3.693)	0.459
IGF2BP3	Low/high (cutoff = 0.1)	2.216 (1.637–2.999)	<0.001	0.727 (0.312–1.694)	0.460
RBM15	Low/high (cutoff = 2.7)	0.682 (0.483–0.963)	0.030	1.523 (0.632–3.668)	0.348
RBM15B	Low/high (cutoff = 5.9)	0.766 (0.563–1.043)	0.091	–	–

*TNM stage, tumor, node, metastasis stage. Italic values represent statistical significance. Bold number means p < 0.05*.

### YTHDF2 Expression Is Associated With Overall Survival With Clinicopathological Characteristics in Clear Cell Renal Cell Cancer

**Figure 1:**
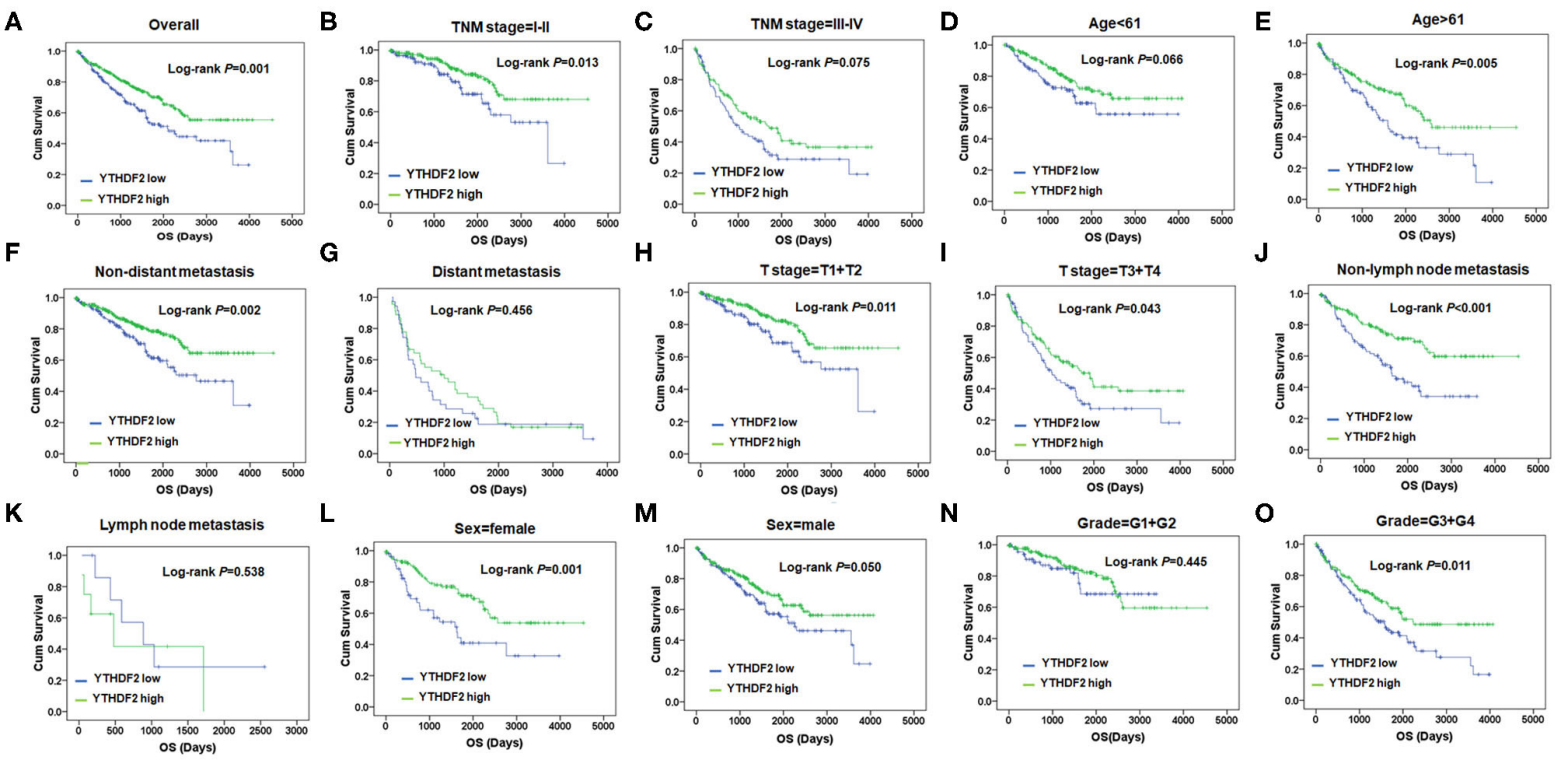
YTHDF2 expression is associated overall survival (OS) with clinicopathological characteristics in clear cell renal cell cancer (ccRCC). **(A)** Kaplan–Meier survival analysis and log-rank test were used to compare differences in OS between the groups classified using cutoff values determined by X-tile; Kaplan–Meier survival analysis and log-rank test were used to analyze the association of YTHDF2 expression and OS stratified by TNM stage **(B,C)**; age **(D,E)**; non-distant metastasis **(F)**, distant metastasis **(G)**; T-stage = T1+T2 **(H)**; and T-stage = T1+T2 **(I)**; non-lymph node metastasis **(J)** and lymph node metastasis **(K)**, sex **(L,M)**; and grade = G1+G2 **(N)** and grade = G3+G4 **(O)**.

Subsequently, the correlation of YTHDF2 mRNA expression and OS was evaluated by log-rank test and Kaplan–Meier survival analysis. As seen in Figure 1, the ccRCC patients with low-level mRNA expression of YTHDF2 presented shorter OS (*P* < 0.001). In stratified analysis (Figures 1B–O), lower YTHDF2 expression was significantly associated with poor prognosis of ccRCC patients with lower TNM stage (stage I–II, *P* = 0.013); elder age (>61, *P* = 0.005); non-distant metastasis (*P* = 0.002); late T stage (T3+T4, *P* = 0.043); non-lymph node metastasis (*P* < 0.001); female gender (*P* = 0.001); and higher histological grades (G3+G4, *P* = 0.011).

### Expression Differences of YTHDF2 in Clear Cell Renal Cell Cancer Samples and Tumor-Adjacent Normal Tissues

**Figure 2:**
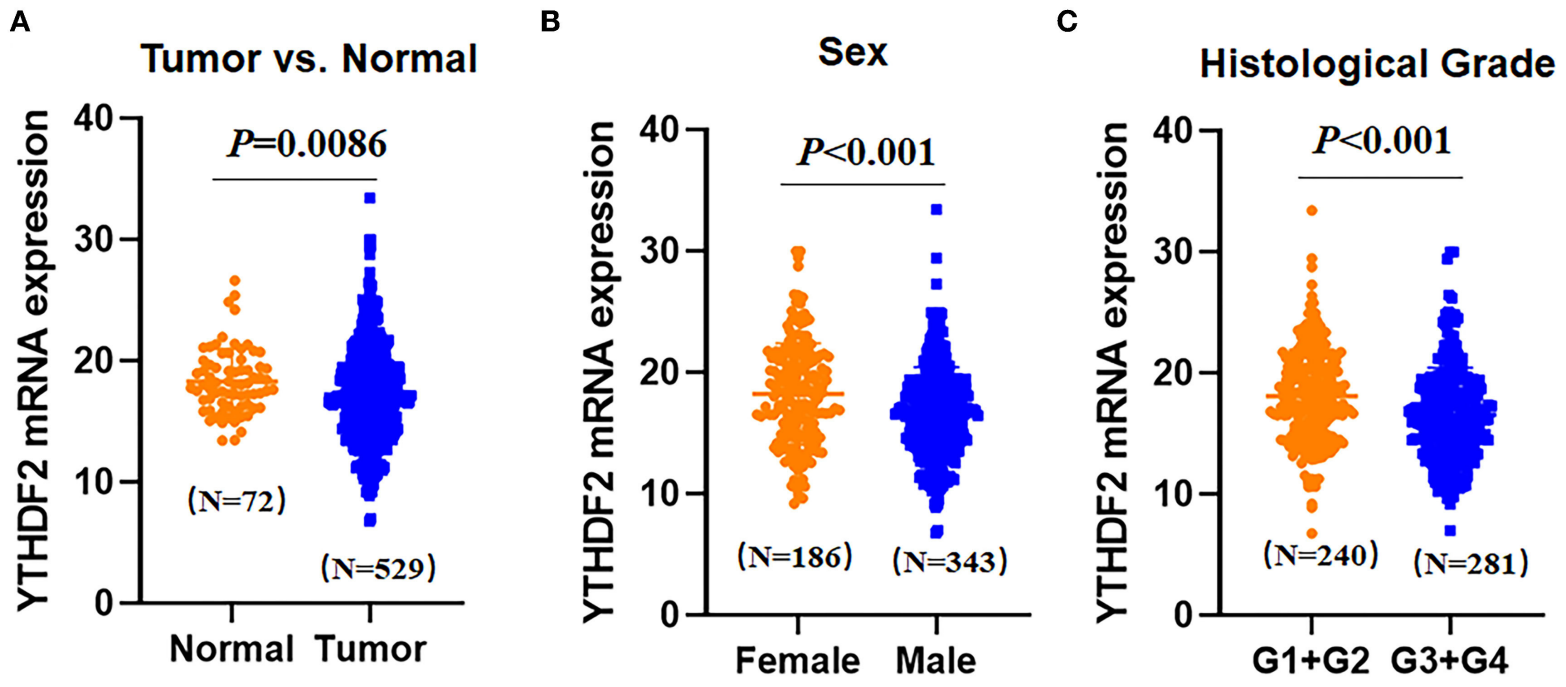
Expression differences of YTHDF2 in clear cell renal cell cancer (ccRCC) samples and tumor-adjacent normal tissues **(A)**. Differential expression of YTHDF2 gene in ccRCC tissues (*N* = 529) and tumor-adjacent normal tissues (*N* = 72); **(B)** differential expression of YTHDF2 in female (*N* = 186) and male (*N* = 343) patients with ccRCC; and **(C)** differential expression of YTHDF2 gene between ccRCC tissues in lower histological grade (G1+G2, *N* = 240) and higher histological grade (G3+G4, *N* = 281).

To analyze YTHDF2 expression in ccRCC and tumor-adjacent normal tissues, we extracted and compared YTHDF2 gene expression from the TCGA database, including 529 ccRCC tumor tissues and 72 tumor-adjacent normal tissues. As shown in Figure 2A, the mRNA expression level of YTHDF2 was evidently decreased in ccRCC (*N* = 529) as compared with tumor-adjacent normal tissues (*N* = 72, *P* = 0.0086). To explore clinical significance of YTHDF2 expression, the correlation of YTHDF2 gene expression and various clinicopathological characteristics of ccRCC were further analyzed. As seen in Table 3 and Figures 2B,C, YTHDF2 expression was significantly lower in male patients (*N* = 186) than female patients (*N* = 343, *P* < 0.001). Meanwhile, YTHDF2 mRNA was significantly less in patients with high histological grade (G_3_+G_4_, *N* = 281) than in patients with low histological grade (G_1_+G_2_, *N* = 240, *P* < 0.001). After the effect of the other clinicopathological characteristics were adjusted for, histological grade (adjusted *P* = 0.010) and sex (adjusted *P* = 0.023) were still significantly associated with YTHDF2 expression in patients with ccRCC.

**Table 3:** Correlation of YTHDF2 expression and clinicopathological variables.

**Clinical features**	**Patients (*n* = 529)**	**YTHDF2**		***p-*value**	**Adjusted *p*-value**
		**Low expression (0–15.8)**	**High expression (>15.8)**		
**Age(years)**					
≤61	281	105	176	0.873	0.892
>61	248	91	157		
**Sex**					
Female	186	54	132	**0.005**	**0.023**
Male	343	142	201		
**T Stage**					
T1+T2	339	122	217	0.499	0.176
T3+T4	190	65	125		
**N stage**					
N_0_	238	92	146	0.369	0.982
N_1_	16	8	8		
**M Stage**					
M_0_	439	154	285	0.138	0.085
M_1_	80	35	45		
**Histological grade**					
G_1_+G_2_	240	68	172	***<0.001***	**0.010**
G_3_+G_4_	281	123	158		
**TNM stage**					
I–II	321	112	209	0.236	0.310
III–IV	205	82	123		
**Cancer status**					
Without tumor	357	126	231	**0.033**	0.429
With tumor	138	63	75		
**Laterality**					
Left	249	97	152	0.363	0.507
Right	279	98	181		
**Race**					
White	458	170	288	0.763	0.237
Asian and Black	64	25	39		
**Hemoglobin**					
Normal	183	61	122	0.162	0.640
Low	261	104	157		
**Platelet**					
Normal	358	128	230	0.334	0.596
Low	44	19	25		
**Serum calcium**					
Normal	150	59	91	0.517	0.807
Low	203	73	130		
**WBC**					
Normal	266	101	165	0.451	0.778
Elevated	163	56	107		

*TNM stage tumor, node, metastasis stage. Italic values represent statistical significance. Bold number means p < 0.05*.

### Identification of YTHDF2-Regulated Multiple Pathways in Clear Cell Renal Cell Cancer by Gene Set Enrichment Analysis

**Figure 3:**
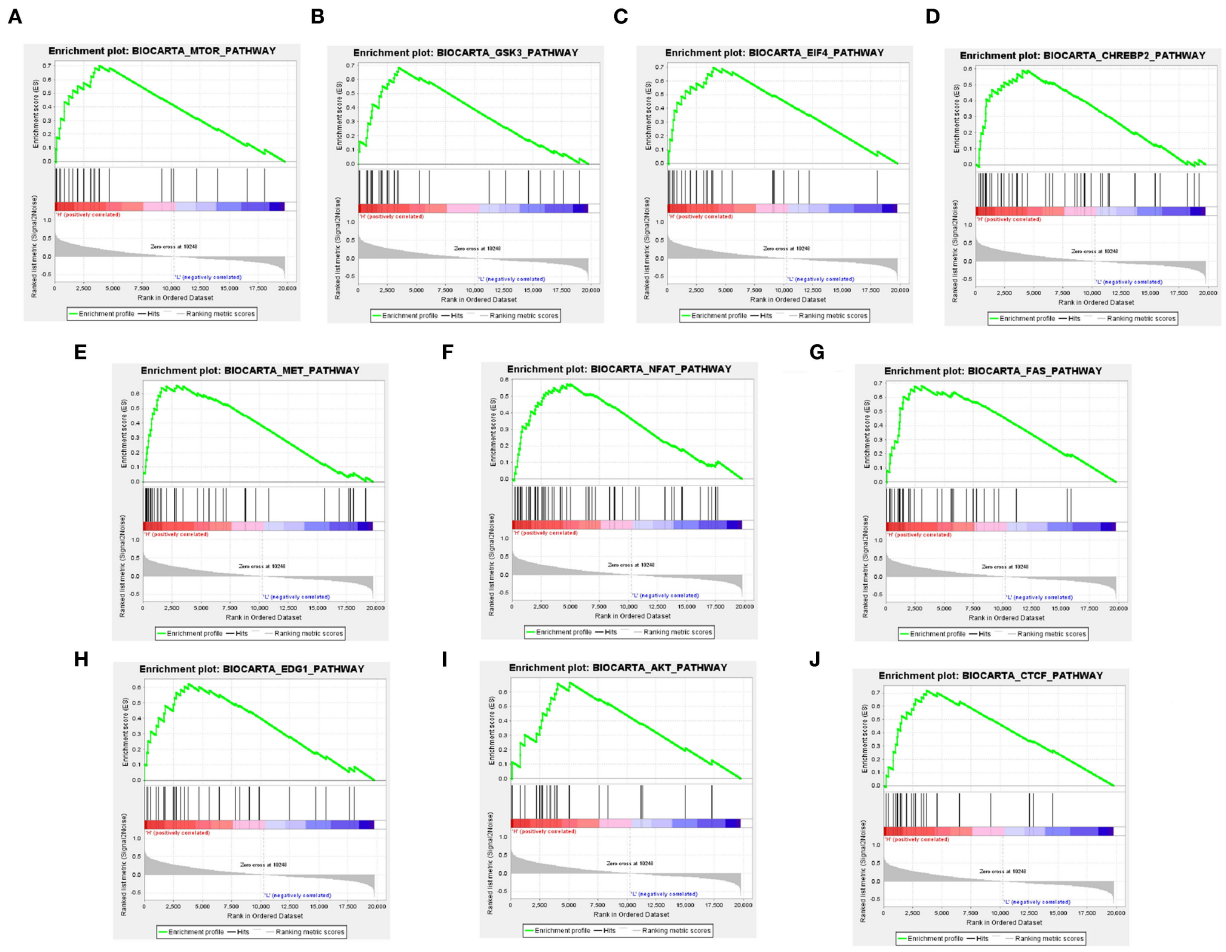
Identification of YTHDF2-regulated multiple pathways in clear cell renal cell cancer (ccRCC) by Gene Set Enrichment Analysis (GSEA). GSEA results showing mTOR pathway **(A)**, GSK3 pathway **(B)**, EIF4 pathway **(C)**, CHREBP2 pathway **(D)**, MET pathway **(E)**, NFAT pathway **(F)**, FAS pathway **(G)**, EDG1 pathway **(H)**, AKT pathway **(I)**, and CTCF pathway **(J)** are differentially enriched in YTHDF2 increased expression phenotype. ES, enrichment score; NES, normalized ES; NOM, normalized *P*-value.

To investigate the alteration of YTHDF2-related pathways in ccRCC, GSEA was performed between two data sets with low- and high-YTHDF2 expression. GSEA showed that there are significant differences in enrichment of MSigDB Collection (NOM *P*-val < 0.05, FDR < 0.05). According to their NES, the top 10 most evidently enriched pathways were screened (Figure 3). It shows that mTOR pathway, GSK3 pathway, EIF4 pathway, CHREBP2 pathway, MET pathway, NFAT pathway, FAS pathway, EDG1 pathway, AKT pathway, and CTCF pathway were significantly enriched in the YTHDF2-related phenotype.

## Discussion

Herein, we discovered that several specific m6A-related genes were closely related to distinct OS and that YTHDF2 can serve as an independent risk factor in ccRCC. What is more, our result showed that YTHDF2 mRNA expression significantly correlated with histological grade and sex. Therefore, YTHDF2, a key m6A-related gene, could serve as a prognostic biomarker and a therapeutic target of ccRCC.

m6A was initially reported by Ronald Desrosiers in 1974 (22), but the precise mechanism and regulatory function of the m6A modification remained largely unknown until recently (23). The m6A modifications were manipulated by precise interplay of recognition, removal, and deposition regulators, and increasing evidences indicated that m6A regulator contributed to initiation and progression of tumors (24). Noticeably, the biological role of these m6A regulators can be variable relying on the disease. For instance, Liu et al. (25) found that high METTL3 expression and decreased regulation of METTL14, METTL16, FTO, and ALKBH5 were positively correlated with poor prognosis in RCC patients. Zhou et al. (26) reported that YTHDF1 served as an independent poor prognostic factor in hepatocellular carcinoma (HCC). Wu et al. found that METTL3, METTL14, WTAP, and FTO present a valuable predictive strategy for breast cancer and contribute to the development of breast cancer (27). To date, the relationship of m6A-regulated genes and ccRCC prognosis is not clear. Herein, we provided an overall summary of the roles of m6A in tumorigenesis of ccRCC. Analysis of the TCGA-KIRC database suggested that elevation of METTL3, IGF2BP1, IGF2BP2, and IGF2BP3 and decreased expressions of ALKBH5, FTO, METTL14, YTHDF2, YTHDC1, YTHDC2, ZC3H13, METTL16, KIAA1429, CBLL1, and RBM15 in ccRCC were associated with poor OS probability. Furthermore, results showed that YTHDF2 could be an independent prognostic factor affecting OS. Similar results were previously reported. Specifically, Li et al. (19) observed that METTL3 exerted an oncogene role in RCC. Zhuang et al. (28) reported that FTO inhibits ccRCC via FTO-PGC-1α pathway. These m6A-related genes could be potential biomarkers utilized clinically in diagnostic and prognostic capacity for ccRCC.

YTHDF2 has been identified as an m6A-binding protein and regulating stability of mRNA (29). YTHDF2 accelerates degradation of target mRNAs through recognizing and binding with m6A sites in 3′-UTR (30, 31). Researchers demonstrated that YTHDF2 played key roles in the cancer progression. Zhong et al. (32) found that YTHDF2 targeting MEK/ERK pathway impacted on growth of HCC cells. Sheng et al. found that YTHDF2 caused tumor growth through altering 6PGD mRNA translation in lung cancer (33). Another study (34) found that YTHDF2 was overexpressed in pancreatic cancer and related to patients' poor prognosis. Kidney cancer is characterized by metabolic disorders (35). Several recent studies reported that YTHDF2 played an important role in the regulation of lipid metabolism (36, 37). However, the role of YTHDF2 in ccRCC metabolic disorders remains unknown. The present study analyzed the association of YTHDF2 with several metabolic-related factors by using TCGA-ccRCC data, including 6-phosphogluconate dehydrogenase (6-PGD) (33). As the result show in Figure S1, there is a weak positive correlation of YTHDF2 and 6-PGD in human ccRCC (Pearson correlation = 0.181, *P* < 0.001, *N* = 529). It is possible that alteration of YTHDF2 expression impacted on 6-PGD and cancer metabolic-related pathways in human ccRCC. These findings indicated that YTHDF2 may act as a potential diagnostic and prognostic biomarker for cancer. The present study found that decreased YTHDF2 expression had poor OS in ccRCC patients with lower TNM stage, higher age, non-distant metastasis, non-lymph node metastasis, female gender, and higher histological grade.

GSEA indicated that multiple cancer signaling pathways (mTOR pathway, GSK3 pathway, EIF4 pathway, CHREBP2 pathway, MET pathway, NFAT pathway, FAS pathway, EDG1 pathway, AKT pathway, and CTCF pathway) were differentially enriched in YTHDF2 increased expression phenotype. Previous studies reported that mTOR pathway (38), GSK3 pathway (39), AKT pathway (40), EIF4 pathway (41), MET pathway (42), NFAT pathway (43), and FAS pathway (44) acted as essential factors in the development of renal cancer and that CHREBP2 pathway (45), EDG1 pathway (46), and CTCF pathway (47) were also cancer-related pathways. Therefore, we supposed that poorer prognosis of ccRCC mediated by decreased YTHDF2 may be related to these pathways directly or indirectly, while the association YTHDF2 expression with these pathways and the precise mechanism need clarification.

There are still limitations in our study. First, the detailed reasons of death in patients cannot be obtained from the TCGA data portal; disease-free survival (DFS) was not considered. Second, the correlation between protein expression of m6A regulator and ccRCC prognosis is not clear. Third, the precise mechanism of the m6A regulator impact ccRCC patient prognosis has not been addressed. Thus, substantial proofs are needed to translate these results into clinical benefit.

In summary, our results demonstrated that downregulation of YTHDF2 (a key m6A-related gene) is associated with poor prognosis of ccRCC patients, suggesting that YTHDF2 can serve as a prognostic biomarker of ccRCC. In addition, GSEA showed that YTHDF2 impacted on multiple cancer signaling pathways, including mTOR pathway, GSK3 pathway, EIF4 pathway, and CHREBP2 pathway. Our findings provide a novel strategy for treatment of ccRCC through regulating epigenetic modification of target genes.

## Data Availability Statement

The datasets presented in this study can be found in online repositories. The names of the repository/repositories and accession number(s) can be found in the article/Supplementary Material.

## Author Contributions

ZM, DD, MS, LL, and BH analyzed and interpreted the data. BH and NW wrote the manuscript. All authors contributed to the article and approved the submitted version.

## Conflict of Interest

The authors declare that the research was conducted in the absence of any commercial or financial relationships that could be construed as a potential conflict of interest.

## Supplementary Material

Figure S1 The correlation of YTHDF2 and 6PGD in ccRCC.
