# Supplementary figures and images for: Prognostic Value of YTHDF2 in Clear Cell Renal Cell Carcinoma
Source: Front Oncol. 2020 Sep 23;10:1566. doi: 10.3389/fonc.2020.01566 (PMC7546891; doi:10.3389/fonc.2020.01566)

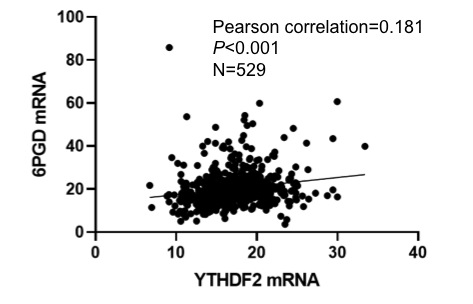

Supplement: Figure S1 — The correlation of YTHDF2 and 6PGD in ccRCC. [file Image_1.JPEG]
